# Atypical Mycobacterium Skin Infection in a Heart Transplant Patient

**DOI:** 10.7759/cureus.72887

**Published:** 2024-11-02

**Authors:** Daniel Calkins, Margarita R Cabrera Cancio, Cynthia A Mayer, Benjamin Mackie

**Affiliations:** 1 Infectious Diseases, University of South Florida Morsani College of Medicine, Tampa, USA; 2 Transplant Infectious Disease, Infectious Disease Associates of Tampa Bay, Tampa, USA; 3 Infectious Diseases, Citrus Memorial Hospital, Inverness, USA; 4 Cardiology, Tampa General Hospital, Tampa, USA

**Keywords:** atypical mycobacterium, heart transplant, immunosuppression complications, mycophenolate mofetil, non-tuberculosis mycobacterium

## Abstract

Atypical mycobacterium infections are an uncommon cause of cutaneous infection, and they are especially rare infections of the facial region. Immunocompromised patients, such as transplant patients, are at higher risk for infections of this nature with concurrent hematogenous spread to other organ systems. We report a patient with a previous heart transplant who developed an atypical mycobacterium infection of the skin with possible dissemination to the lung. The case outlines the difficulty in diagnosing this condition along with the challenges of prescribing an effective drug regimen to treat this infection. Azithromycin, minocycline, rifabutin, and high-dose ethambutol were effective in clearing the skin and lung lesions.

## Introduction

Atypical mycobacterial infections are caused by non-tuberculous mycobacteria (NTM), which are environmental organisms commonly found in soil, water, and contaminated medical equipment and can cause skin infections, lymphadenitis, and pulmonary infections [1]. They have peptidoglycan and characteristic mycolic acid in their cell walls that can be identified with acid-fast staining and the presence of multi-nucleated giant cells histologically [1]. The presentation of an atypical mycobacterial skin infection is often erythematous papules or a rash that can progress to ulcers and disseminated infection. The patients most at risk are the immunocompromised and those who have had recent surgical procedures [2]. Here, we discuss a heart transplant patient on immunosuppression who developed difficult-to-diagnose facial lesions.

## Case presentation

A 68-year-old male with a past medical history of hypothyroidism, coronary artery disease, and heart transplant presented to Tampa General Hospital with a history of multiple erythematous plaques with surface erosions and crusting on the left cheek and right forehead with tan-colored drainage. The patient had a history of ischemic cardiomyopathy and underwent placement of a left ventricular assist device and later a heart transplant on March 14, 2016. He was cytomegalovirus (CMV) positive, and his initial immunosuppression included tacrolimus, prednisone, and mycophenolic acid. In 2017, he was treated for a cutaneous mycobacterium infection of the left upper arm, suspected to be *Mycobacterium marinum*, that resolved with minocycline and ethambutol. *Mycobacterium marinum* was suspected due to a patient history of frequent contact with fish along with granulomatous inflammation and dermal fibrosis on biopsy. However, this was not confirmed because cultures were negative.

**Figure 1:**
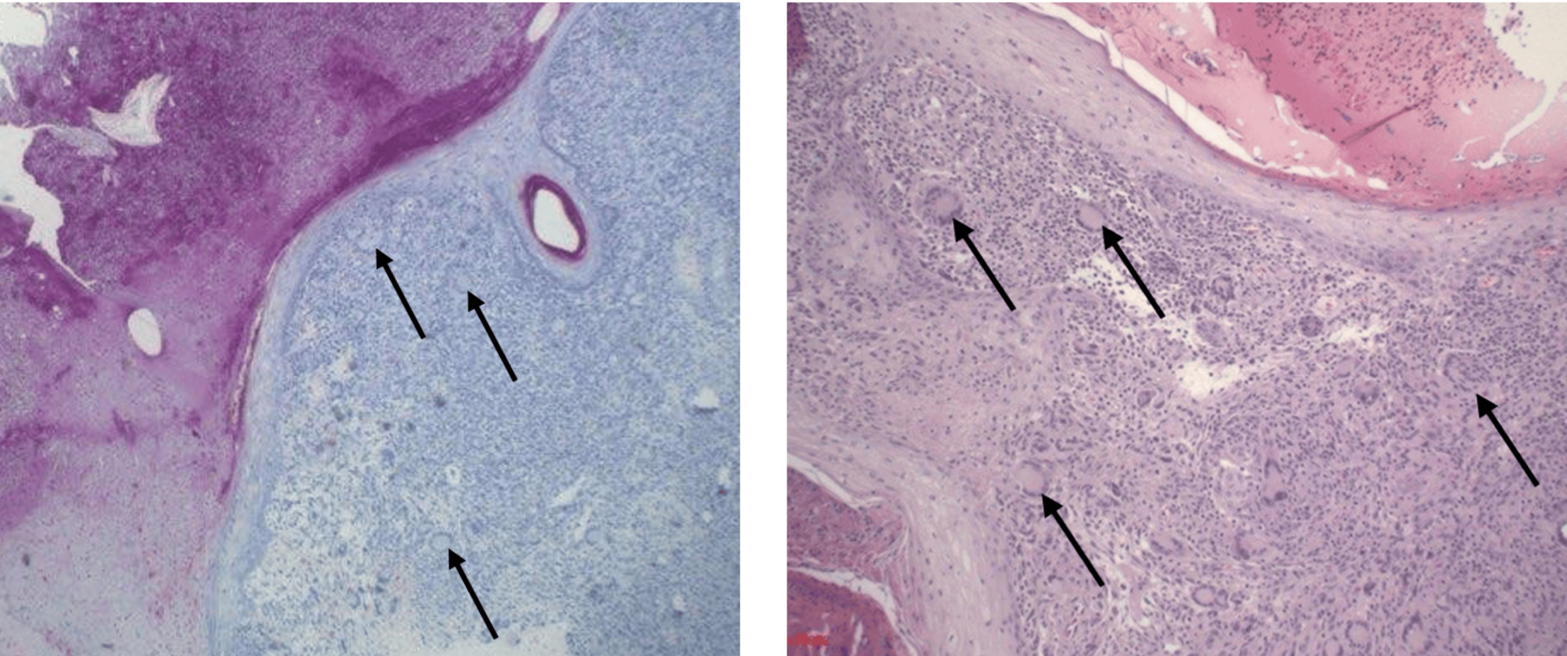
Histopathology of skin biopsies Arrows represent caseating granulomas with multinucleated giant cells

In December 2019, he developed a painful, burning rash on the right side of his forehead, which progressed to involve the left cheek and the inferior left periauricular region. The rash originated as minor erythematous plaques and progressed in size with associated exfoliation and tan-colored drainage. The patient denied fever, chills, night sweats, hemoptysis, and weight loss but endorsed dyspnea. Dermatology performed multiple skin biopsies, demonstrating granulomatous dermatitis with abundant dermal multinucleated giant cells and caseous necrosis (Figure 1). Initial treatment consisted of Plaquenil and intralesional steroids, with the subsequent addition of ketoconazole cream. Response to therapy was poor, and he was referred to a local infectious disease specialist for further evaluation. In July 2020, further workup included a chest CT scan, revealing diffuse patchy peripheral opacities, which could be consistent with a possible atypical mycobacterial infection. Four years prior, he had a negative chest CT scan. A bronchoscopy with bronchoalveolar lavage (BAL) was performed, which was negative for an infectious etiology. Additional skin biopsies for routine, fungal, and acid-fast bacilli (AFB) cultures were negative, and blood cultures, PCR (polymerase chain reaction), and serum QuantiFERON Gold were negative. Serum angiotensin-converting enzyme (ACE) levels were normal, suggesting that the changes to the lung were not due to sarcoidosis. He was treated with azithromycin and ethambutol with initial improvement but later had progression of the rash.

**Figure 2:**
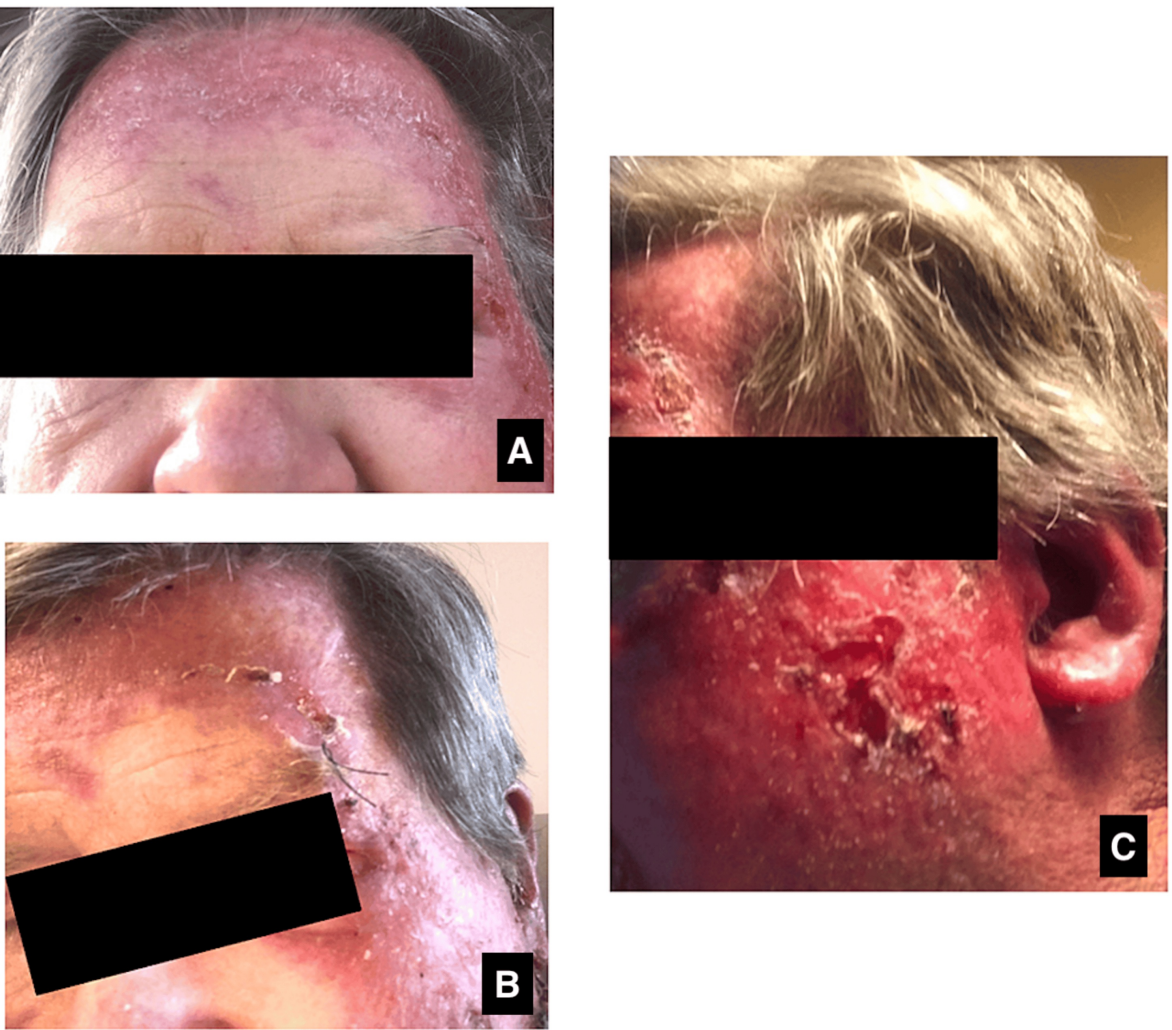
Skin rash of the face displaying eroded plaques and patches (A) Erythematous patch of the right forehead; (B) erythematous plaques with erosion extending down the left forehead and cheek; (C) erythematous plaques with erosion of the left cheek and preauricular region

In October 2020, the patient was admitted to Tampa General Hospital for a persistent/progressive, painful, burning rash on his right forehead that extended down the left side of the face (Figure 2) and was seen by Transplant Infectious Disease and Dermatology. The patient denied chest pain, cough, dyspnea, and all other symptoms. The physical exam revealed a well-developed, well-nourished male in no acute distress with lungs clear to auscultation, normal heart sounds, and normal bowel sounds. There were dull, erythematous, eroded patches and plaques across the forehead extending down the left temple. Labs revealed an elevated C-reactive protein (CRP) of 0.26 mg/dL and an erythrocyte sedimentation rate (ESR) of 16 mm/hour, which is within the reference range. A biopsy of the lesion was performed, along with a repeat chest CT scan. The chest CT scan again demonstrated peripheral patchy airspace disease, bronchial wall thickening, and reactive mediastinal lymphadenopathy, which was concerning for atypical mycobacterium lung infection. Another biopsy of the facial lesions showed giant cells and patchy necrosis. The AFB smear was positive; however, routine fungal and AFB cultures were again negative. Blood samples and bronchial washings were again collected, and mycobacterial PCR tests targeting *Mycobacterium fortuitum*, *Mycobacterium chelonae*, and *Mycobacterium abscessus* were completed and returned negative. The bronchoalveolar lavage (BAL) showed no significant growth and was negative for AFB and fungi, including the mycobacterial PCR testing. Notably, *Mycobacterium haemophilum* could not be ruled out from bacterial cultures because of iron supplement requirements and lower growth temperatures [3]. QuantiFERON Gold testing was negative, as was extensive testing for other non-infectious causes of the skin lesions. Specifically, antinuclear antibody (ANA), proteinase Ab, myeloperoxidase Ab, rheumatoid factor, and ACE level were all negative. These tests included cultures that did not grow anaerobic bacteria, fungi, or AFB; gram-stain smear, methenamine silver stain, periodic acid-Schiff (PAS), CD10 and CD68, and Congo red stain were negative. Azithromycin 250 mg and ethambutol 800 mg were prescribed; minocycline 100 mg was added due to a history of possible *Mycobacterium marinum* in the past. The facial lesions initially improved with this regimen; however, several weeks later, his rash worsened. Subsequently, rifabutin 300 mg was added, and ethambutol dosing was increased to 1600 mg daily. Antibiotics were continued for 24 weeks.

**Figure 3:**
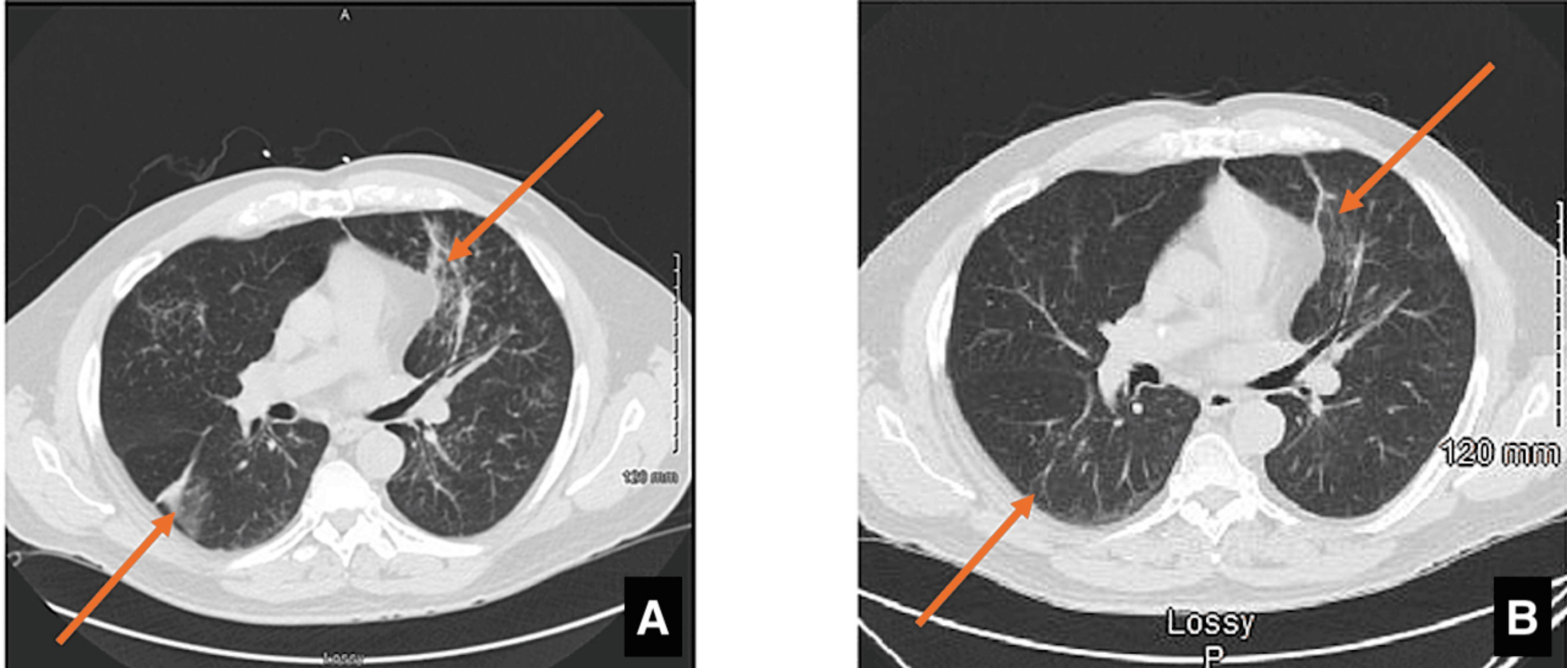
Lung CT scan (A) CT scan with perihilar bronchial wall thickening, reactive nodes, and diffuse ground-glass peripheral opacities; (B) CT scan after antibiotic therapy

The patient had a slow but consistent improvement in the facial rash that was fully resolved by April 2021 after a six-month course of antibiotic therapy. During the course of his treatment, he also developed an eosinophilia that was initially believed to be a drug reaction; however, stool testing showed the presence of an amoeba infection. The erythema subsided, as well as the scaling of the skin, and the lesions were replaced with scar tissue. A follow-up chest CT scan for comparison showed significant improvement in the ground-glass opacities and resolution of reactive nodes, suggesting clearing of the infection (Figure 3). A chest CT scan performed a year later was completely normal. Since the last patient encounter regarding the facial rash with Infectious Disease in April of 2021, there have been no signs of recurrent infection.

## Discussion

Atypical mycobacteria are uncommon causes of cutaneous infections. They should be suspected when the patient displays a persistent skin rash such as cellulitis, macules, ulcers, or abscesses unresponsive to conventional treatment and with no other explainable cause. The most common sites of involvement are the knees, feet, hands, and elbows; however, infection of the face and spread to the lungs is unusual [4,5]. It is also important to note that clinical judgment and ruling out of other pathogens are necessary for an accurate diagnosis because false-negative results can occur, as was seen in this case [6]. Specific growth requirements for AFB cultures, such as the requirements for *Mycobacterium haemophilum*, limit the efficacy of cultures. Diagnostic testing from a skin biopsy or wound culture includes PCR analysis, staining, and culture for acid-fast organisms, emphasizing the appropriate mycobacterial cultures to identify mycobacterium species [7].

According to a study published in the Journal of Clinical Tuberculosis and Other Mycobacterial Diseases, there have been previous cases in which a patient displayed a positive AFB smear and negative mycobacterium species PCR; in these cases, 96% of patients had NTM infections [8]. Although PCR is more sensitive than AFB smear, technical issues such as suboptimal PCR primers, sample mycobacterial DNA degradation, and low bacterial load may have contributed to the negative PCR findings.

The optimal treatment for cutaneous mycobacterial infections is complex and challenging. Once the species has been identified and sensitivities are available, a multi-drug regimen is generally warranted. Effective drugs can include azithromycin, rifampin, rifabutin, ciprofloxacin, ethambutol, and amikacin, but they are species-specific. The changes seen on the chest CT scan in July 2020 were ultimately resolved after treatment with antibiotics, and this could be suggestive of the dissemination of atypical mycobacterium skin infection to the lungs.

The process of atypical mycobacterium dissemination has been documented in immunocompromised patients; however, there is no literature at this time that specifically describes spread to the lungs, which could make this a novel finding [9,10]. Unfortunately, due to the negative BAL, this could not be confirmed. Due to the rare nature of the location and presentation in our patient and the lack of positive cultures or PCR testing of both skin biopsies and BAL fluid, the diagnosis was difficult to make, and a broad-spectrum, multi-drug regimen was used, which successfully resolved the infection. A biopsy of the skin did finally show an AFB on staining. These findings, in combination with a high index of suspicion, led to successful treatment of his condition. Finally, many other non-infectious causes of granulomatous dermatitis were ruled out on extensive testing.

Immunosuppressive drugs are critical in preventing organ rejection in solid organ transplant patients. However, one of the risks associated with these drugs is an increased susceptibility to opportunistic infections such as *Aspergillus* species, Epstein-Barr virus (EBV), and *Mycobacterium* species [1]. For instance, tacrolimus functions by binding the FK506 binding protein, thus inhibiting calcineurin phosphatase and reducing *IL-2* gene expression. Mycophenolate reduces T and B cell lymphocytes by inhibiting inosine 5'-monophosphate dehydrogenase. The resultant effect of these drugs is decreasing the response of the immune system to foreign antigens present in donor organs, therefore decreasing the likelihood of transplant rejection but also decreasing an appropriate response to infectious agents. Interestingly, our patient's course was also complicated by eosinophilia, initially thought to be from a medication but was later found to be due to an amoebic infection. This was confirmed with stool PCR and was treated with metronidazole. This condition further highlights these patients' risk for additional infections.

## Conclusions

This case demonstrates the difficulty in obtaining a diagnosis of atypical mycobacterial infections. Facial involvement is uncommon, and the organism and specific species were not identified despite extensive testing. However, empiric therapy was warranted in this case, and the patient had a successful outcome with the resolution of his facial rash and the abnormal chest CT findings with no further recurrences. It is not confirmed whether the skin infection spread to the lungs due to the negative BAL; however, it is critical to note that a second chest CT scan showed resolution of the pathology after treatment with anti-mycobacterial antibiotics. It is important to rule out unusual causes of cutaneous infection in immunocompromised patients and to treat when there is a high level of suspicion despite definitive positive results.
